# Co-design knowledge mobilization tools for universal accessibility in municipalities

**DOI:** 10.3389/fresc.2024.1331728

**Published:** 2024-07-01

**Authors:** Maëlle Corcuff, Marie-Eve Lamontagne, François Routhier, Ernesto Morales

**Affiliations:** ^1^ Center for Interdisciplinary Research in Rehabilitation and Social Integration, Centre Intégré Universitaire en Santé et Services Sociaux de la Capitale Nationale, Québec, QC, Canada; ^2^ School of Rehabilitation Sciences, Faculty of Medicine, Université Laval, Québec, QC, Canada

**Keywords:** co-design, implementation, universal accessibility, municipal organization, disability

## Abstract

**Introduction:**

Modern research teams are re-evaluating conventional methods with the aim of improving the usefulness of knowledge for users, focusing on the role of knowledge users in shaping innovation. In disability field, encouraging participatory research inherently involves diverse perspectives and inclusion, which aligns with the principles of universal accessibility. By actively involving individuals with various backgrounds, abilities, and needs in the research process, we can better understand and address the challenges faced in adopting universal accessibility. This approach ensures that solutions are more comprehensive, inclusive, and effectively cater to the needs of all individuals, fostering a more equitable and accessible environment for everyone. Despite municipal organizations mandating universal accessibility action plans, they lack tools for efficient implementation. The aim of this study was to develop knowledge mobilization tools tailored to a specific municipal context in Quebec, Canada, to facilitate the implementation of universal accessibility measures by municipal employees.

**Methods:**

The co-design process employed in this study was organized into four distinct stages, following the Morales model: (1) Exploration (2) Co-Design (3) Validation (4) Development.

**Results:**

Stages one and two highlighted the employees' lack of awareness about universal accessibility issues and their need to have more information and resources about how universal accessibility is encountered in their work. A steering committee co-designed three video vignettes about universal accessibility, the city's action plan and measures included in it.

**Discussion:**

The co-design approach used in this study allowed us to observe the non-linear nature of partnership research with an organization as complex as a municipality. Our study shows significant advantages of collaboration between the municipal sector and research.

## 1 Introduction

In response to the scarcity and delays in implementing research results, research teams are reassessing their methodologies to improve the accessibility and usability of knowledge for end-users. One approach to accelerate the adoption of knowledge is the use of participatory research paradigms that highlight the significance of actively engaging knowledge users in knowledge generation process and implementation of innovative solutions (1–4). Many studies have shown that involving knowledge users in the knowledge creation process significantly and positively impacts the implementation of innovation (5, 6). It also positively influences individual changes in knowledge, attitudes, and beliefs (7–9). The benefits of engaging stakeholders in the implementation process have been supported in the knowledge production process (4, 7, 10). For example, it enables stakeholders to contribute to the design and implementation of accessibility measures. This collaboration ensures that solutions meet the specific needs of users (7). It also promotes acceptance and compliance with accessibility measures. Collaborating with local community organizations increases awareness and acceptance of accessibility initiatives, leading to higher compliance rates and sustainable results.

There is a significant importance in advocating for the utilization of participatory research and involving stakeholders such as decision-makers, municipal actors, disabled people, or others working in the disability field to address, among other things, universal accessibility of social and physical environments issues. Participatory research is seen by researchers and partners as a relevant method for accelerating and promoting the adoption of universal accessibility measures, thereby ensuring equitable social participation and rights for Persons with Disabilities (PWDs) or marginalized populations and fostering their independent engagement in various face ts of society. Universal accessibility refers to the character of a product, process, service, environment, or information that, within an equity vision and through an inclusive approach, enables any individual to engage in activities independently and achieve equivalent outcomes (11). In this project, we focused on the accessibility of the physical and social environment.

Municipal organizations play a leading role in developing universal accessibility solutions in urban contexts. With the adoption of the Convention on the Rights of Persons with Disabilities (CRPD), municipalities have been at the forefront of implementing best practices in universal accessibility (12, 13). In the province of Quebec, Canada, municipalities of more than 15,000 citizens must develop universal accessibility action plans (12, 14), which are policies detailing specific measures for planning project organization activities (15). Despite their mandate to draw up a universal accessibility action plan, municipal organizations currently face a lack of knowledge mobilization tools and strategies to facilitate the implementation of the diverse measures outlined in these plans by the decision-makers, managers and employees (16).

This can be attributed to contextual factors (e.g., recent emphasis on universal accessibility issues, large number of universal accessibility measures and administrative rules) (17) and complexities lying within municipal organizations (e.g., multiple hierarchy levels, staff retention, large number of employees and administrative units, diversity of actors and stakeholders) (9, 17–19). By 2050, it is estimated that more than two-thirds of the world's population will live in urban areas (20). With an increasing number of urban dwellers and a substantial growth of global population of individuals with disabilities (21), active involvement of municipal employees in the implementation of universal accessibility measures holds significant benefits and empowers them to contribute to a more inclusive and equitable society (22, 23). Haynes et al. (24) demonstrated that in the implementation of partnerships to strengthen policy, knowledge mobilization activities used to foster engagement, capacity building and partnership formation yielded positive results, and that co-design could be strengthened by greater sharing of the decision-making process.

In response to this challenge, a city in the province of Quebec (Canada) has recognized the need to engage in a co-design process to develop knowledge mobilization tools and strategies that will optimize the implementation of universal accessibility practices. Co-design aims for active participation and integration of users' points of view throughout the design process (25). As such, co-design seemed entirely appropriate for this partnership research, where the involvement of researchers and municipal stakeholders is equally important at each step of the project. Besides, the co-design approach in organizations helps the stakeholders to realize their project goals (26). It provides individuals with more direct involvement in defining their needs and priorities and collaboratively finding solutions, influencing decisions, and achieving better outcomes (27).

This study took place in a large tourist city in the province of Quebec, with a population of around 550,000 citizens and a metropolitan community of about 840,000 people. This municipal organization has 5,000 full-time employees working as managers, civil servants, professionals, technicians, workers, or seasonal workers in 33 administrative units. The City has involved 25 of the city's administrative units to varying degrees in the universal accessibility action plan. In each of the administrative units involved in the action plan, an employee is designated as responsible for universal accessibility. The City's internal Universal Accessibility Committee brings together the 25 employees responsible for each unit. These employees are members of each team who have volunteered for this role and who have developed expertise in universal accessibility through their training or experience. This municipal organization was selected for this study based in its needs to develop tools, and because of the lack of scientific and practical knowledge about the organization's internal context, which may or may not allow the implementation of universal accessibility measures. It's also a large city with significant needs in terms of universal accessibility. Indeed, it has a complex hierarchical organization and significant needs due to its aging population and the heritage character of its environment (28, 29).

In that context, the main objective of this study was to develop knowledge mobilization tools tailored to a specific municipal context to facilitate the implementation of universal accessibility measures by municipal employees. The secondary objective was to address the relevance of a co-design method in a municipal organizational context.

## 2 Methods

**Figure 1:**
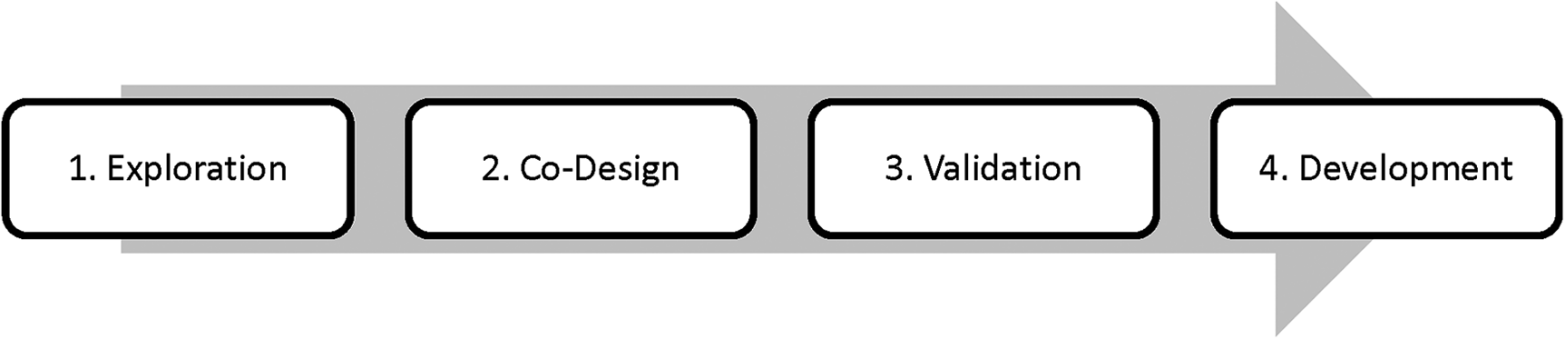
Morales’ model of co-design process.

Co-design is a participatory methodological process that facilitates the solution development through collaboration between various stakeholders, such as researchers and partners. Co-design has an individual, social and material dimension that encourages the creative process and facilitates multi-professional negotiation by transcending restrictions (30). The research process employed in this study was organized into four distinct stages, following the model proposed by Morales et al. (31): (1) Exploration (2) Co-Design (3) Validation (4) Development (see Figure 1). The exploration phrase aims the better understand the problem and the participant's experience. The co-design phase is to promote creative thinking among the team to design a solution. The validation phase is to evaluate pertinence and feasibility of the ideas and development aims to translate the results into a tool or object (31). The co-design process was carried out with stakeholders from the municipal organization who are acknowledged by their peers for their expertise and interest in universal accessibility.

### 2.1 Exploration

The exploration step employed an experience-centered approach i.e., to explore users’ experience to better grasp the problems to be solved (25). It enabled the gathering of participants' experiences and perceptions regarding the context. This information was significant because the subsequent stages of the process were built upon the data obtained in this phase. The co-design phase was subsequently grounded in a genuine need expressed by the individuals directly affected by the proposed solution. During exploration stage, our data collection was conducted through the combination of survey and focus groups.

#### 2.1.1 Survey

A survey adapted from the Consolidated Framework for implementation research (CFIR) (32) allowed to collect information and insights regarding the barriers and facilitators of implementing universal accessibility measures. To create this survey, we conducted a rapid literature review (33) of the utilization of the CFIR determinants (34) in assessing implementation in organizational settings. CFIR is a framework widely used in implementation, which combines 19 theories of implementation from various disciplines. It is made up of 39 constructs groups under 5 domains: intervention characteristics, outer setting, inner setting, characteristics of individuals and process (34, 35). This framework offers a common language about facilitators and barriers of implementation and supports implementation of evidence-based practices from design to evaluation (19). It recently has been used for policies interventions and fits well to evaluate complex interventions such as urban social policies (19). This process facilitated the identification of survey questions tailored to the specific municipal context, as recommended by Nilsen and Bernhardsson (36). Three authors (MC, MEL, FR) and two other experts in knowledge mobilization and universal accessibility carried out this process. A preliminary version of the survey was presented to the municipal partners. Three iterations were required to ensure that the questions were customized for the local context and easily understandable for municipal employees. The changes made were clarifications of questions to ensure that the language would be clear and well understood by all employee respondents.

The questionnaire consisted of 30 Likert-type questions and 5 briefs open-ended questions. The number of questions that needed to be answered depended on the response to the initial question in the survey, which inquired about the respondents' familiarity with the municipal organization action plan. This first question was important because of the low level of knowledge of the action plan reported by this city's municipal employees according to our previous study (16). If respondents were familiar with the action plan, they had to answer an additional 10 questions, bringing the total to 40 questions. The questionnaire was distributed to all municipal employees via mass email. Two follow-up emails were sent. The results of the questionnaire were analyzed using frequency distribution, for the Likert-type questions (37) and content analysis to extract meaningful insights and themes for the qualitative questions (38, 39).

#### 2.1.2 Focus groups

Three focus group sessions of 120 min each were conducted. The aim of these focus group sessions was to have a better grasp of all participants’ experiences and perceptions regarding universal accessibility in a municipal context, along with complement the information gathered from the questionnaires. These focus groups involved employees of seven administrative units, selected based on their responses to the questionnaire and their active engagement in universal accessibility initiatives within the city. A discussion guide was prepared, drawing from the questionnaire results, to delve deeper into the survey findings. The questions aimed at gathering more information on the actions taken in universal accessibility, the information and tools they needed to facilitate the implementation of universal accessibility measures, and the content and format of these tools. Additional questions were incorporated to comprehensively document the diverse needs identified. The discussions were recorded for transcription. An inductive thematic analysis (38–40) was done by the first author. The coded results were then discussed within the research team and with partners. At this stage, validation of the analysis with methodological experts from the research team (MEL, FR) and with the partner improved the rigor of the analysis process.

### 2.2 Co-design

The second phase of our co-design process aimed to stimulate creative thinking among partners and research team members (31). To initiate this co-design phase, a steering committee was established, consisting of five employees dedicated to promoting universal accessibility within their respective administrative units. A preparatory meeting was convened to provide context of the project and to present the findings from the survey and focus groups. Over the course of six months, the committee had six meetings of two hours. These meetings were held virtually through the Teams platform and were recorded. The committee engaged in discussions regarding the format, content, and modalities of the knowledge mobilization tools. Following each meeting, the research team made improvements to the tools suggested, which were then presented with modifications and discussed in the subsequent meeting. These iterative cycles enabled the adaptation of prototypes tools tailored to the specific context and needs of the city.

### 2.3 Validation

The third step involved presenting the prototypes that emerged from the co-design sessions with the steering committee to a broader audience of municipal employees, which was all members of the City's universal accessibility committee. The goal was to confirm whether the tools effectively addressed the needs and barriers commonly experienced by their colleagues, the translation process into formal tools, along with the feasibility of their potential implementation. To achieve this, the stage 2 outcomes of the Morales model were shared with the City's internal committee on universal accessibility, composed of twenty-five employees responsible for universal accessibility within their administrative units. Notes were taken during the meeting. The discussions held in this step served to validate the appropriateness and relevance of the co-designed tools and provided all the necessary input to proceed with the final development of these tools and their subsequent implementation.

### 2.4 Development

The final step involved translating the prototypes into formal tools that would be used within the administrative units. Considering the municipal organizational context, the implementation strategy of the knowledge mobilization tools, including the prototypes developed by the steering committee and the research team, was initially presented to the directors of the administrative units. This step allowed the managers to gain clarity regarding the expectations for their employees and to better understand the relevance of these tools. The implementation process started within three pilot units, namely Heritage and Culture, Communication, and Citizen Engagement. These teams were selected as they corresponded to the respective teams of three members of the steering committee. This enabled faster pre-testing and better feedback. To ease the implementation of the tools, an instructional guide was developed and presented to the three responsible for universal accessibility of the pilot units. This animation guide, available in a printed or not word format, was used to facilitates the presentation of the tools during team meetings and supported discussions around universal accessibility among their colleagues.

## 3 Results

### 3.1 Exploration

#### 3.1.1 Survey

##### 3.1.1.1 Participants

Employees from all administrative units completed the survey (*n* = 277; response rate = 32%). They had different types of job: officials (56%), professionals (30%), equipment manager (7%), other (7%). Nearly half of the employees had been working in their current position for more than 5 years (47%).

##### 3.1.1.2 Implementation barriers

Regarding universal accessibility measures, the results showed that employees find universal accessibility principles complex (69%) and hard to implement (76%). They also have difficulty seeing the adaptability of the measures to their reality as municipal employees (60%). Concerning outer setting, more than half (53%) of employees said they needed to better understand who universal accessibility measures were for and who they would serve in the community. Regarding inner setting, employees said they lack the resources and tools to implement universal accessibility measures (59%). In terms of individual characteristics, knowledge and beliefs were identified as a barrier. Employees said they knew what universal accessibility was (64%), but they barely knew that the city had a universal accessibility action plan (67%) and did not receive a presentation of this action plan (77%). Also, they didn't know what role universal accessibility played in their work or administrative unit (40%). As for the process, the results of the survey highlighted that employees were unaware if their manager has planned to implement universal accessibility measures within the unit (63%). They also didn't know what was planned to execute (71%) and evaluate (94%) universal accessibility measures in their team.

##### 3.1.1.3 Tools needed

The results of the inductive thematic analysis brought out specific needs for tools. Visual information tools such as videos, documents or infographics about universal accessibility, the action plan and specific measures for each unit was the most mentioned need (40%). Other needs mentioned included a short and specific guide about universal accessibility actions in their daily work (15%), toolbox with references (13%), checklist (10%) and sensitivity trainings (9%). Some employees also mentioned more specific needs such as identification of the responsible for universal accessibility in their unit (3%), adapted equipment for citizens (2%), or dedicated budget for universal accessibility measures (0.7%). Only 2% of the people mentioned having all the tools they needed. For example, the recreation, culture and events teams reported a greater need for specialized equipment, or a budget allocated to accessibility, given their proximity to citizens. On the other hand, employees in teams further away from citizens (e.g., Finance, Human Resources) were more interested in identifying the person responsible for universal accessibility in their team, so that they could refer to the right person.

#### 3.1.2 Focus groups

The three focus groups were held with respectively 6, 4 and 4 participants (*n* = 14) from 7 administrative units (Event management, Office of Major Events, Citizen Engagement, Human Resources, Communications, Records and Archives, and Culture Heritage).

##### 3.1.2.1 Facilitators

Regarding universal accessibility measures, employees had a good perception of the implementation of universal accessibility measures. They mentioned the benefits of universal accessibility measures for them and their colleagues as being themselves citizens. Other facilitators to implementation were underlined toward inner setting. Developed networks and communication with other organizations and with citizens, a positive learning climate within the municipal organization, relative priority to improve implementation of universal accessibility measures shared by participants and leadership engagement of their team's manager were reported as helping them to prioritize this issue. For the characteristics of individuals, all participants reported self-efficacy and positive attitudes towards universal accessibility measures. Finally, representatives of administrative units in universal accessibility [champions] and consultations or partnerships with PWDs during activities was highlighted as process facilitator in the implementation of universal accessibility measures.

##### 3.1.2.2 Barriers

Barriers were highlighted by participants of all groups. First, when considering intervention attributes, employees are raising concerns about the absence of adaptability to their tasks or unit, limited trialability within certain teams, and the complexity of universal accessibility measures. Second, the lack of knowledge about external policies and incentives or about what are the people with disabilities' needs is reported as an outer setting barrier by employees. Readiness for implementation has been tackled as an inner setting barrier, due to the lack of available resources. The implementation climate was also discussed as barrier because of the impact of pandemic (use of virtual mode) and work overload due to staff shortage (learning climate), the relative priority and the access to knowledge and information. Regarding characteristics of individuals, knowledge and beliefs were underlined by 3 participants as a barrier because of false beliefs or stereotypes about universal accessibility. Participants also mentioned they perceive that their organization talks about accessibility without being accessible for their own employees. Process was not discussed as a barrier in the focus group.

##### 3.1.2.3 Needs

When asked to discuss the different tools that employees might need, employees stated aspects related to the content and format of these tools. Participants named testimonials of PWDs as having a significant impact on their awareness. Guides and resources on universal accessibility about how to plan an accessible event or activity, or how to answer special needs of a city employee were also mentioned. For format, sensitive training, short video clips, intranet toolbox and informal discussions around coffee break with colleagues were discussed. There was no consensus on the preference between virtual or face-to-face activities.

Table 1 shows results of survey and focus group classified according to the CFIR domains as facilitators or barriers to the implementation of universal accessibility measures by municipal employees. This synthesis of facilitators and barriers served as a starting point for the subsequent codesign phase.

**Table 1:** Facilitators and barriers to implementation of universal accessibility measures by municipal employees according to the CFIR.

CFIR domains	Facilitators		Barriers	
	Item	CFIR construct associated	Item	CFIR construct associated
Intervention characteristics	Benefits for them or colleagues	Relative advantage	Absence of adaptability to their tasks or unit	Adaptability
	Positive perception of universal accessibility measures	Need of evidence strength and quality	Complexity of universal accessibility measures	Complexity
Outer setting	Developed networks and communication with other organizations and with citizens	Cosmopolitanism	Lack of available resources or information	Individuals needs and resources
	Representatives of administrative units in universal accessibility [champions]	Peer pressure		
	Action plan	External policies and incentives		
Inner setting	Developed networks and communication with other organizations and with citizens	Network and communications	Readiness of change by the work teams	Readiness for implementation
Characteristics of individuals	Self-efficacy	Self-efficacy	False beliefs or stereotypes about universal accessibility; lack of knowledge on universal accessibility or on action plan	Knowledge and beliefs about intervention
	Positive personal attitudes with universal accessibility and their responsibility	Individual identification with organization		
Process	Leadership engagement of their team's manager	Engaging	Unaware if their manager has planned to implement universal accessibility measures	Planning
			Do not know what was planned to execute	Executing
			Do not know what is evaluated	Reflecting and evaluating

### 3.2 Co-design

The co-design sessions followed the exploration phase. A total of seven meetings were necessary to complete the co-design process. During the initial session, the research team and the steering committee collaborated to categorize and prioritize the various tools and barriers identified during the exploration phase. Given the project deadlines, the committee opted to initiate the co-design process by creating three video vignettes. The development of videos seemed to be appropriate to the research context since they can convey a general idea such as universal accessibility more easily and clearly than other media. They also facilitate retention of the knowledge we aimed to convey since they respond to the three main principles of knowledge translation according to Bennet and Jessani (41), i.e., the presentation of solid, accessible and contextualized knowledge, through relational dialogue and exchange, and based on a skills base of researchers and knowledge users creating opportunities for knowledge translation. The purpose of these videos was to raise awareness among municipal employees on universal accessibility, in addition to addressing the major barriers identified (lack of information, knowledge, need for a deeper understanding of how universal accessibility affects employees in their work).

The subsequent six meetings of the steering committee followed an iterative process between the research team and the steering committee regarding the content of the videos. Two sessions were dedicated to each video vignette. During the first session, an initial scenario was proposed by the research team, with the integration of scientific content. This proposal was subjected to feedback and critique from the steering committee members. Their suggestions for modifications were considered to tailor the content to their specific municipal context. Subsequently, the research team revised the scenario, incorporating the received feedback, and presented a second version during the second session. This second version was then reviewed and validated by the steering committee. This iterative process was repeated three times, corresponding to the creation of the three video vignettes. These six sessions were spaced out over six months, with one meeting occurring each month.

### 3.3 Validation

The modified version of the content of the video vignettes, based on the results of the co-design phase, was presented at a meeting of the City's internal universal accessibility committee. This committee validated that the content of the videos was meeting the identified needs and was suitably customized to the context. They also gave further explanations to ensure a comprehensive understanding of the mandates for all universal accessibility units so the development and implementation would be adapted to realities. They were able to understand their role in the further implementation of the video within their respective units.

### 3.4 Development

A professional videographer was engaged to shoot the videos, for a high-quality production. For the first video clip, the cast included both a city employee and a person with an intellectual disability (PWD). The PWD received financial compensation for their participation in the first video capsule, and all actors involved in the project signed consent forms. In the second video clip, two city employees took on the acting roles, and for the final video, a total of 8 employees participated in the filming process. The videos had durations ranging from 3 to 5 min each. Here's a brief overview of the content and style of each video:

• **First Video:** This video introduces the concept of universal accessibility and illustrates how it is applied to city services.
• **Second Video:** The second video presents the city's action plan and outlines the various measures planned by the municipality to promote universal accessibility.
• **Third Video:** In the third video, employees share personal accounts of how universal accessibility is integrated into their work and discuss their accomplishments in this domain. This video takes on a more direct and storytelling style, with participants speaking directly to the camera.

Each video production required half a day to a full day of shooting to ensure the content was well-crafted. To disseminate the different video capsules within the administrative units, an implementation strategy is currently developed collaboratively by the research team and the steering committee. This strategy will undergo validation by the relevant departments within the organization. Following this, an evaluation strategy will be implemented in three test units (Heritage and Culture, Communication, and Citizen Engagement) to fine-tune the implementation approach for subsequent administrative units. This all-encompassing approach will enable us to integrate the videos as effectively as possible in the workplace, and to achieve the desired objectives.

## 4 Discussion

This study reported a participatory process that aimed to create knowledge mobilization tools for municipal employees to facilitate the implementation of universal accessibility measures outlined in their action plan. In this research, we used Morales and colleagues’ co-design methodology within a collaborative partnership in a municipal organization context. Utilizing a co-design approach in collaboration with a complex entity like a municipality underscores the need for tailored knowledge mobilization strategies to engage various stakeholders throughout the implementation process.

Our study illustrated that the process employed allowed us to create tools and develop an adaptable implementation strategy aligned with specific needs and context. Our results differ from those reported by Dubois et al. (42) who argument that co-design requires collective action and effective organization of the environment and is not optimal in a complex context. Our research illustrated that it is possible to employ a participatory-based co-design approach in intricate organizational settings such as municipalities. However, we still don't know what exact characteristics of the context facilitates or hinders the co-creation process. Even though we know some of context characteristics of this City, they have not been measured. We can make assumptions about it with the use of the CFIR framework, but they are not empirically supported. We can therefore assume that we are still lacking tools that are well known and well shared, so that they can be put to good use. It would be interesting the measure the different characteristics, so we can draw more solid conclusions. Although the municipal organization is complex and highly hierarchical, there seems to be a coherence in opinions and needs shared by employees. It is possible that, in different contexts, organizational changes during a co-design process could rather diminish the efficiency of the process, prevent such validation of results and limit generalizability. For example, authors demonstrated that co-design in a healthcare organizational context presented significant challenges due to organizational resistance to change, and the need for change in culture, behaviors, time, resources, and managerial support within the organization (43). We believe that the weight of municipal policies regarding universal accessibility also helped counter this resistance to change and released resources to facilitate the process. In fact, although few employees were aware of their administration's universal accessibility action plan, the existing policy on this subject enabled us to use it as a lever for change to demonstrate the importance of this issue. It allows for a certain momentum, fostering the commitment of municipal stakeholders in the process. This also justifies our relevance in developing tools that promote understanding of this action plan and the implementation of the measures outlined therein.

Following the linear four-stage process based on the Morales (31) model, we observed that within a municipal organization, the process naturally became more iterative due to the multiple validations required at every stage. In the context of partnership research, these iterations at different stages showed greater significance as they impact positively partner engagement and involvement levels throughout the project, ultimately influencing the implementation process. These iterations also enabled a more natural diffusion of the process within the organizational structure, following the organization's habitual ways of communicating. The iterative nature of the co-design process appears to be recurring in studies on design. Indeed, Steen (44) emphasize that it is normal, and even beneficial, for the co-design process to be iterative, ensuring alignment between needs and responses. Other studies in the field of disability, which have conducted co-design studies or processes, have also highlighted the relevance of these iterations to ensure a comprehensive understanding among all stakeholders and to address the needs of the population involved (45, 46).

Emmons and Chambers (47) emphasized the importance of applying implementation science strategies to social and urban policies to enhance our ability to address health-related social determinants. They underscored research's role in understanding why intervention succeed in specific context. Integrating the CFIR framework into data collection and analysis in our study provided deeper insights into the connection between results and the implementation of universal accessibility measures, particularly within municipal context. Labbé et al. (19) also highlighted CFIR's relevance in municipal settings. CFIR's suitability for evaluating complex intervention, like universal accessibility measures, supported our partnership research and co-design process, enabling better identification of implementation facilitators, obstacles and stakeholders needs. Contextual understanding is pivotal in implementation science, as it elucidated what works and why (36). According to Nilsen (48), frameworks in implementation science aim to describe and/or guide the process of translating research into practice, understand and/or explain what influences implementation outcomes or to evaluate implementation. Theoretical frameworks, such as CFIR, ensure methodological robustness and mitigate challenges in partnership research. However, their constrained utilization of these frameworks beyond healthcare contexts (49, 50), hinders effective co-design processes across various settings, including patient involvement in service care (51–53).

### 4.1 Strenghts and limitations

This participatory research enabled us to learn more about the co-design process with a municipal partner. Our reflections and conclusions highlight some of the strengths and limitations of our study. Despite the large and diverse group of participants during the exploration phase and the various stakeholders involved in the co-design and validation phases, the survey results, focus group discussions, and co-design deliberations all converged on a common finding. Municipal employees expressed a lack of information, knowledge, awareness, and resources concerning universal accessibility measures. However, it's important to note that this outcome may also be attributed to the co-design process itself. This coherence between the results of the different stages of the research is one of our study's strengths. Indeed, the co-design process made it possible to verify and triangulate the data at the different stages with different methods, and to obtain a saturation of data on the implementation barriers and facilitators of universal accessibility measures by municipal employees. We believe this coherence is mostly attributable to the initial context. Moreover, the flexibility of the process and the various iterations have ensured the development of knowledge mobilization tools adapted to the reality of the context and meeting a real need on the part of knowledge users.

However, we note as a limitation the fact that only one person with a disability was involved in the first video. It would have been interesting to have a person with lived experience on the steering committee, to ensure that the ideas conveyed in the video vignettes reflected the real-life experience of the people concerned. However, we would point out that the primary objective was to reach out to municipal employees, which is why their colleagues were the main participants in the videos.

### 4.2 Future research

The creation of this partnership research aimed to produce usable and transferable results that consider knowledge and expertise of stakeholders (26). Our study highlights significant advantages of collaboration between the municipal sector and research. It seems to facilitate social relevance of research, the creation of tools and interventions better suited to the context, and a higher potential for user engagement with the results. Consequently, these solutions become sustainable and beneficial to the entire community. Partnered research allows for the implementation of more robust and efficient solutions to complex problems. By establishing a partnership between the municipal sector and health research, it becomes possible to develop and implement effective strategies and initiatives that can have a positive impact on the lives of individuals with disabilities. Also, the participation of a large number of municipal employees from diverse backgrounds during the process increased the external validity of our research (40). At times, however, this hampered consensus-building, as we were unable to produce resources that met all needs. So, to begin with, we focused on the needs put forward by the actors involved in the co-design process, based on the needs expressed in the questionnaire and in the focus groups. Finally, we believe that the process used could be replicated in other studies and similar municipal contexts, but that it could also lead to the creation of other tools, according to need.

## 5 Conclusion

The co-design approach used in this study allowed us to observe the non-linear nature of partnership research with an organization as complex as a municipality. In fact, several iterations, and exchanges within the team and with municipal administration departments were necessary to ensure that everyone was comfortable with the process and deliverables. We also note that involving knowledge users in the process from the outset, and at every stage of the project, enables the development of solutions tailored to their real needs. Collaboration between the research team and the municipal organization's team ensured an adapted response to real needs of the municipal partner. Involving knowledge users from the outset and at every stage of the project was extremely beneficial. Their participation ensured the relevance of our research objectives, and their ongoing feedback enabled us to adjust our work. We believe this partnership process reinforced acceptance of the research results since they were able to see the evolution of the project and that their involvement fostered greater confidence in the conclusions reached. Also, by co-creating knowledge, we enriched our understanding of the subject and we learned to overcome some of the obstacles of partnership research, such as adapting to each other's culture and respecting each other's pace.

In the future evaluation of the implementation of co-designed tools, we will assess whether this has had an impact on the knowledge and awareness of municipal employees. We believe that by raising awareness with the video vignettes, employees will feel more sensitive and responsible toward universal accessibility issues. Moreover, this paper highlighted how partnership research and a co-design methodology can be applied with complex partners and complex issues such as knowledge mobilization and universal accessibility.

## Data Availability

The original contributions presented in the study are included in the article/Supplementary Material, further inquiries can be directed to the corresponding author.
